# Clinical outcomes of percutaneous coronary intervention for severely calcified lesions: comparison between the morphologies of severely calcified coronary lesions

**DOI:** 10.1007/s00380-024-02466-7

**Published:** 2024-09-25

**Authors:** Yoriyasu Suzuki, Masahiro Uehara, Hirohiko Ando, Akihiro Suzuki, Akira Murata, Hiroaki Matsuda, Takahiro Tokuda, Tetsuya Amano

**Affiliations:** 1 Department of Cardiology, Aichi Medical University, 1-1 Yazakorimata, Nagakute, Aichi 480-1195 Japan; 2 Department of Cardiology, Nagoya Heart Center, 1-1-14 Sunadabashi Chikusa-ku, Nagoya, Aichi 461-0045 Japan

**Keywords:** Percutaneous coronary intervention, Calcification, Drug-eluting stent

## Abstract

Existing studies evaluating the comparison of clinical outcome of percutaneous coronary intervention (PCI) for severe calcified coronary lesions are limited, and the clinical outcomes of PCI for different morphologies of calcified lesions are controversial. Overall, consecutive 576 lesions with severe calcification that were treated with PCI from 2010 to 2021 at Nagoya Heart Center were investigated. All lesions were assessed using invasive coronary angiogram (CAG) or computed tomography-CAG at 12 months after DES implantation. We divided the patients into three groups based on the results of intravascular ultrasound (IVUS) imaging (concentric calcified lesion [CC] n = 273, eccentric calcified lesion [EC] n = 217, calcified nodule [CN] n = 86). The clinical and angiographic outcomes of each group were investigated retrospectively to compare the prognosis between the three groups and identify predictive factors for the device-oriented composite end points (DoCE). There were no differences in patient characteristics among the three groups, except that there were significantly more patients on dialysis in the CN group. The incidence of DoCE was significantly higher in the CN group than in the other groups (CC; 18.3% vs. EC; 23.5% vs. CN; 36.0%; Log-Rank test; *p* = 0.001). Cox regression analysis showed that the independent predictors of DoCE were CN, insulin use, hemodialysis, right coronary artery lesions, and calcium cracks. The incidence of DoCE was significantly higher in the CN group. Calcium cracks are crucial for improving outcomes in severely calcified lesions, being key predictors of DoCE.

## Introduction

Percutaneous coronary intervention (PCI) is widely used to treat patients with complex coronary artery disease. However, PCI for calcified coronary lesions remains challenging despite significant improvements in drug-eluting stents (DES), atherectomy devices, and techniques. Coronary calcifications are associated with stent under-expansion, greater strut malapposition, and smaller residual lumens after stent deployment, all of which are linked with stent thrombosis and restenosis [1–3]. Calcium fracture during PCI is reportedly associated with better stent expansion [4–6]. Several algorithms that focus on selecting cracking and debulking devices for lesion modification in PCI for severely calcified lesions have also been reported [7–10]. Sakakura et al. reported an algorithm for severely calcified lesions that focuses on the morphology of calcifications [11]. However, few studies have focused on the morphologies of calcifications and compared the clinical outcomes of PCI for severely calcified lesions following these algorithms. This study aimed to investigate the clinical outcomes of PCI using DES for severely calcified lesions and to identify solutions to these issues.

## Methods

### Patients and study design

This retrospective observational study enrolled consecutive patients who fulfilled the following criteria.

1. De novo severely calcified lesions in a native coronary artery.
2. The intravascular ultrasound (IVUS) data before and after DES deployment were available for analysis.
3. With IVUS images that were suitable for analysis.
4. Underwent invasive coronary angiogram (CAG) or computed tomography (CT)-CAG 12 months after DES implantation to assess the patency of treated coronary artery.

**Fig. 1:**
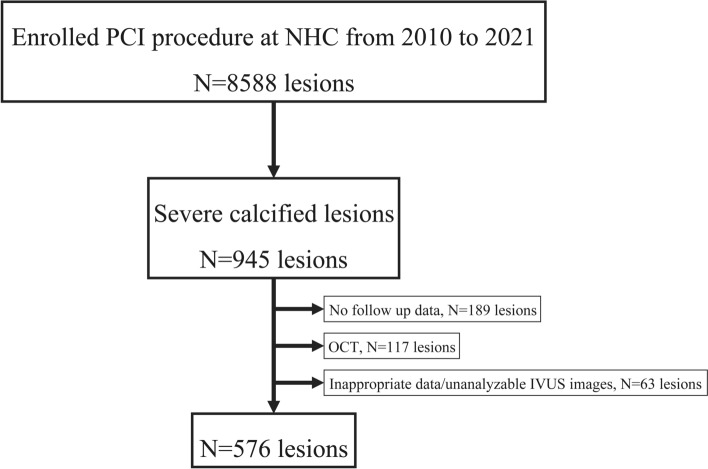
Study flowchart

Lesions that did not meet the above inclusion criteria, such as chronic occlusion lesions, and in-stent restenosis lesions were excluded from this study. The study flowchart is shown in Fig. 1. Between 2010 and 2021, PCI was performed on 8,588 lesions at the Nagoya Heart Center. Of these, 945 were de novo highly calcified lesions; however, 369 lesions were excluded due to inadequate data or having met the exclusion criteria. Therefore, consecutive 576 lesions from 530 patients were included and retrospectively evaluated in this study. Clinical outcomes beyond 12 months from DES implantation were evaluated using medical records and telephone conversations. Written informed consent was obtained from all patients. The study was approved by the ethics committee of Nagoya Heart Center and was conducted in accordance with the Declaration of Helsinki.

**Table 1:** Baseline patient characteristics

	Total (n = 576)	CC (n = 273)	EC (n = 217)	CN (n = 86)	*p*-value
Male, %	76.0	71.1	82.0	76.7	0.018
Age	70.5 ± 8.9	70.8 ± 9.0	70.3 ± 8.7	70.2 ± 9.2	0.787
Clinical diagnosis, %					0.507
SAP	84.9	83.2	87.0	84.9	
OMI	9.4	10.3	8.8	8.1	
STEMI/NSTEMI	3.0	4.4	1.8	1.2	
UAP	2.8	2.2	2.3	5.8	
Prior PCI, %	29.8	29.6	30.4	28.8	0.373
Prior CABG, %	4.7	5.3	4.1	4.7	0.878
HT, %	66.3	66.7	64.5	69.7	0.674
DLP, %	49.8	49.1	52.1	46.5	0.645
DM, %	48.1	49.8	46.1	47.9	0.679
Insulin use, %	6.4	6.6	6.0	6.9	0.94
CKD, %	52.9	54.9	46.5	62.8	0.025
HD, %	25.0	24.9	19.8	38.3	0.003
PAD, %	23.3	24.5	21.2	24.4	0.659

*SAP* stable angina pectoris, *OMI* old myocardial infarction, *STEMI* ST-elevated myocardial infarction, *NSTEMI* non- ST-elevated myocardial infarction, *UAP* unstable angina pectoris, *HT* hypertension, *DLP* dyslipidemia, *DM* diabetes mellites, *CKD* chronic kidney disease, *HD* hemodialysis, *PAD* peripheral artery disease

The primary endpoint of this study was defined as the device-oriented composite end points (DoCE) according to the ARC-2 consensus document [12].

### Interventional procedure, image analysis, and definition

Aspirin and a P2Y12 inhibitor, such as clopidogrel or prasugrel, were administered in all patients as pretreatment, and weight-adjusted heparin was administered to maintain an activated clotting time of > 300 s. The sequences of PCI and DES selection depended on the operator’s discretion and the patient’s coronary anatomy. Data on the presence of coronary calcifications based on fluoroscopy assessments were analyzed. Severe calcification was defined as radiopacities observed without cardiac motion before contrast injection that usually affected both sides of the arterial lumen [3].

IVUS was conducted before and after balloon dilatation using a commercially available 40- or 60-MHz IVUS system. The IVUS catheter was advanced distally to the target lesion, and imaging retrograde to the aorto-ostial junction at an automatic pullback speed of 0.5 mm/s was performed. If the IVUS catheter did not pass through the lesion initially, the atherectomy device was first used to modify the calcified lesion, then the IVUS catheter was promptly inserted for intravascular evaluation. Two independent cardiologists (Y.S and M.U) assessed all IVUS data. IVUS assessments were conducted according to the American College of Cardiology Clinical Expert Consensus Document on Standards for Acquisition [13], Measurement and Reporting of Intravascular Ultrasound Studies using validated software (QIVUS, Medis Medical Imaging Systems, Leiden, Netherlands). Cross Sects. (1 mm) of IVUS images obtained immediately before DES implantation and after PCI were analyzed. The definition of the IVUS findings is shown below.

**Fig. 2:**
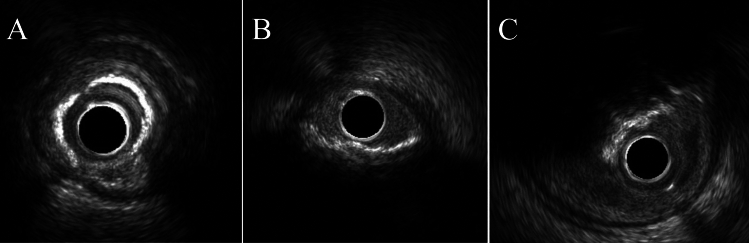
Typical IVUS images of target lesions. **A** concentric calcified lesion, **B** eccentric calcified lesion, **C** calcified nodule lesion

**Fig. 3:**
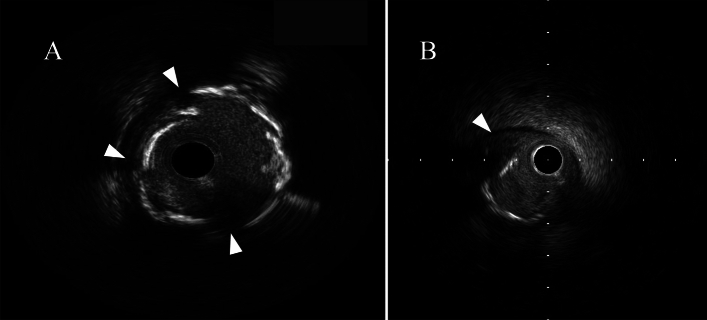
Typical IVUS images after lesion preparation. **A** calcium crack (arrow), **B** calcium shoulder dissection (arrow)

1. All calcium arcs were measured relative to the center of mass of the lumen at minimum lumen area. The maximum calcium arch grade at the target lesion was as follows: Concentric calcification was defined as a calcium arch ≥ 270° (CC group). Eccentric calcification was defined as a calcium arch < 270° (EC group). A CN was defined as a convex shape of the luminal side of calcium (Fig. 2 illustrates the typical IVUS image of each group) [14].
2. A calcium crack was defined as a gap of calcium and direct exposure of calcium to the lumen at the gap (Fig. 3A illustrates the typical IVUS image).
3. The dissections at the shoulders of the calcification were defined as calcium shoulder dissection (Fig. 3B illustrates the typical IVUS image).
4. Stent malapposition was defined as ≥ 1 stent struts separated from the vessel wall.
5. The stent symmetry index was calculated as the minimum stent diameter divided by the maximum stent diameter (as measured at each cross-section) [15].

We divided the patients into three groups based on the morphology of calcified lesions on IVUS (concentric calcified [CC] lesion n = 273, eccentric calcified [EC] lesion n = 217, calcified nodule [CN] n = 86) groups.

**Table 2:** Baseline angiographic characteristics and used PCI devices

	Total (n = 576)	CC (n = 273)	EC (n = 217)	CN (n = 86)	*p*-value
Target lesion, %					< 0.0001
LAD	55.0	61.2	60.4	19.8	
LCx	9.5	7.3	14.3	4.7	
RCA	32.1	28.9	20.7	70.9	
LMT	2.8	2.2	3.7	4.6	
Moderate-severe bending, %	24.1	24.2	21.2	31.4	0.093
PCI device					
OA, %	8.9	7.3	7.4	17.4	0.01
RA, %	73.1	73.3	74.7	68.6	0.582
POBA, %	34.2	27.5	43.3	32.7	0.009
SBA, %	65.8	72.5	56.7	67.4	0.001

*LAD* left anterior descending coronary artery, *LCx* left circumflex coronary artery, *RCA* right coronary artery, *LMT* left main trunk, *OA* orbital atherectomy system, *RA* rotational atherectomy, *POBA* plain old balloon angioplasty, *SBA* scoring balloon angioplasty

### Statistics

Continuous data are presented as mean ± standard deviation. Comparisons of continuous variables among the three groups were analyzed using one-way analysis of variance. Discrete variables are expressed as counts and percentages, which were assessed using the chi-square test. Time-to-event data were summarized as Kaplan–Meier estimates according to the different morphologies of severely calcified coronary lesions and were compared using the log-rank test. The Cox regression hazard model was used to identify the risk of DoCE. In all analyses, *p* < 0.05 was considered statistically significant. All statistical analyses were conducted using SPSS version 26 (IBM Corp., Armonk, NY, USA).

## Results

In approximately 60% of the lesions, the IVUS catheter initially failed to pass through the target lesion and required ablation with atherectomy devices before IVUS assessment. The median follow-up period after DES implantation was 35.4 months (IQR; 17.8–63.5 months).

### Baseline patient characteristics (Table 1)

Except for the significantly greater number of patients with CKD on hemodialysis (HD) in the CN group (CKD: CC; 54.9% vs. EC;46.5% vs. CN; 62.8%, respectively, p = 0.025, HD: CC; 24.9% vs. EC;19.8% vs. CN; 38.3%, respectively, p = 0.003), there were no significant differences in patient characteristics among the three groups.

**Table 3:** Results of Quantitative coronary artery analysis and Quantitative intravascular ultrasound analysis

	CC (n = 273)	EC (n = 217)	CN (n = 86)	*p*-value
Reference vessel diameter, mm	2.69 ± 0.57	2.57 ± 0.69	3.14 ± 0.69	< 0.0001
Post MLD, mm	2.79 ± 0.52	2.67 ± 0.45	3.13 ± 0.64	< 0.0001
Acute gain, mm	1.86 ± 0.58	1.82 ± 0.56	1.90 ± 0.61	< 0.0001
Total stent length, mm	30.9 ± 13.1	31.6 ± 13.7	25.5 ± 12.7	0.001
Stent type, %				0.213
CoCr-EES	39.9	42.4	35.6	
PtCr-EES	35.5	34.0	33.7	
ZES	12.5	13.4	12.1	
Others	12.1	10.2	18.6	
Number of stent	1.2 ± 0.4	1.2 ± 0.9	1.1 ± 0.3	0.310
Stent size, mm	2.98 ± 0.43	2.91 ± 0.39	3.36 ± 0.44	< 0.0001
Minimum stent area, mm^2^	6.37 ± 2.30	5.33 ± 2.53	6.81 ± 2.01	0.027
Minimum stent symmetry index	0.76 ± 0.08	0.61 ± 0.07	0.69 ± 0.08	< 0.0001
Mean stent symmetry index	0.85 ± 0.03	0.76 ± 0.03	0.82 ± 0.03	0.027
Calcium crack, %	77.3	8.3	4.7	< 0.0001
Calcium shoulder dissection, %	49.5	87.8	48.8	< 0.0001
Stent malappostion, %	33.3	53.9	83.7	< 0.0001

*MLD* minimum lesion diameter

### Angiographic findings, Target lesion location, and PCI devices (Table 2)

In the CN group, the target lesion location was mostly the right coronary artery (RCA) (70.9%), whereas the left circumflex artery was significantly more common in the EC group (14.3%). Orbital atherectomy (OA) was performed more frequently in the CN group (17.4%) and scoring balloon angioplasty (SBA) was less frequently performed in the EC group (CC; 72.5% vs. EC; 56.7% vs. CN; 67.4%, p = 0.001, respectively).

### The results of QCA and QCU (Table 3)

The reference vessel diameter and acute gain were significantly smaller in the EC group. Calcium cracks were rarely observed in both EC and CN groups (CC;77.3% vs. EC; 8.3% vs. CN; 4.7%, p < 0.001, respectively). The frequency of calcium shoulder dissection was significantly higher in the EC group (CC;49.5% vs. EC; 87.8% vs. CN; 48.8%, p < 0.001, respectively). Stent malapposition was observed more frequently in both EC and CN groups (CC;33.3% vs. EC; 53.9% vs. CN; 83.7%, p < 0.001, respectively). QCU analysis revealed that the minimum stent area (MSA) was significantly smaller, and the stent symmetry index (SSI) was significantly lower, in the EC group.

**Table 4:** Clinical outcomes

	CC (n = 273)	EC (n = 217)	CN (n = 86)	*p*-value
Initial Success, %	100	99.5	100	0.437
Coronary perforation, %	0.4	0.9	1.3	0.160
Subacute thrombosis, %	0.7	0.5	0	0.704
TLR at 12-month, %	5.1	12.4	14.0	0.005
Binary restenosis, %	13.6	19.8	31.4	0.001
Myocardial infarction, %	4.4	2.8	11.6	0.005
All cause death, %	10.6	12.6	8.1	0.621
Cardiovascular death, %	5.9	5.5	3.5	0.691

*TLR* target lesion revascularization

### The clinical outcomes (Table 4 Figure 4)

In all three groups, the success rate of the initial procedure was approximately 100%, and there were almost no complications. At 12 months after DES implantation, the incidence of TLR was highest in the CN group followed by the EC and CC groups (CC;5.1% vs. EC; 12.4% vs. CN; 14.0%, p = 0.005, respectively).

**Fig. 4:**
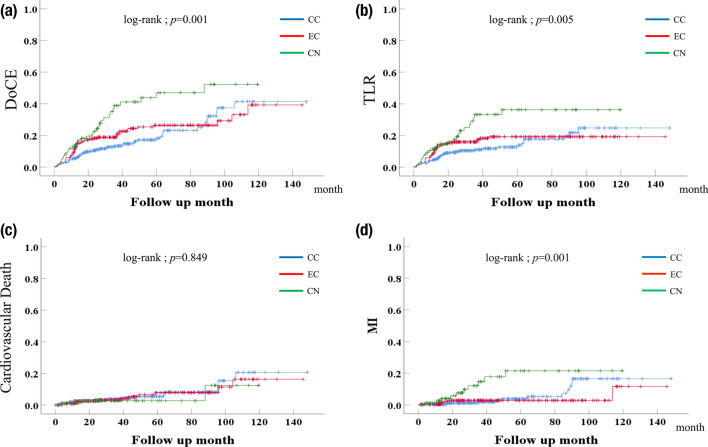
Kaplan–Meier curve of each clinical outcome according to the different morphologies of severe calcified coronary lesions. **A** incidence of DoCE: demonstrates a significantly higher incidence of DoCE in the CN group compared to CC and EC groups (CN: 36.0%, CC: 18.3%, EC: 23.5%; Log-Rank test; p = 0.001). **B** incidence of TLR: shows the incidence of TLR at 12 months post-DES implantation, with the CN group having the highest TLR rate (CN: 14.0%, EC: 12.4%, CC: 5.1%; p = 0.005). **C** incidence of cardiovascular death: illustrates the incidence of cardiovascular death among the three groups, with no statistically significant differences observed between the CN, CC, and EC groups. **D** incidence of MI: presents the incidence of MI across the groups, showing a similar trend with no significant differences between the CN, CC, and EC morphologies

The incidence of DoCE was significantly higher in the CN group (Fig. 4), while that in the CN group continued to increase over time 12 months after DES implantation. Meanwhile, the incidence of cardiovascular death was not different among the three groups.

### ***Comparative analysis between patients with and without DoCE (***Table 5)

**Table 5:** Comparison between the patients with and without DoCE

			Univariate		Multivariate	
	DoCE (-) N = 460	DoCE ( +) N = 116	OR (95%CI)	*p*-value	OR (95%CI)	*p*-value
CN, %	12.2	25.9	2.518 (1.525–4.152)	0.001	1.806 (1.035–3.154)	0.038
Male, %	73.4	87.1	2.458 (1.376–4.391)	0.001	1.198 (0.716–2.006)	0.491
Insulin user, %	4.3	14.7	3.778 (1.909–7.404)	< 0.0001	1.764 (1.035–3.008)	0.037
HD, %	17	56.9	6.465 (4.160–10.046)	< 0.0001	4.318 (2.896–6.438)	< 0.0001
PAD, %	20	36.2	2.270 (1.459–3.533)	0.001	1.099 (0.883–1.363)	0.397
RCA lesion, %	26.1	56	23.611 (.369–5.505)	< 0.0001	3.207 (2.118–4.857)	< 0.0001
Calcium crack, %	43.5	29.3	0.539 (0.347–0.837)	0.006	0.657 (0.420–0.928)	0.036
Calcium edge dissection, %	66.1	61.2	0.810 (0.532–1.233)	0.329		
Stent malappostion, %	44.8	63.8	2.172 (1.426–3.309)	< 0.0001	1.259 (0.823–1.926)	0.289
OA, %	9.6	6	0.607 (0.266–1.385)	0.276		
RA, %	72.8	74.1	1.070 (0.673–1.701)	0.816		
SBA, %	66.7	62.1	0.816 (0.535–1.244)	0.499		
MSA < 5.5mm^2^, %	35.4	43.1	1.760 (1.167–2.655)	0.028	1.138 (0.767–1.687)	0.521
Rota burr size, mm	1.76 ± 0.23	1.71 ± 0.22		0.863		
Total stent length, mm	30.1 ± 12.3	35.4 ± 18.1		< 0.0001		
Stent size, mm	3.00 ± 0.44	3.06 ± 0.45		0.402		
Post MLD, mm	2.86 ± 0.58	2.78 ± 0.53		0.274		
Acute gain, mm	2.12 ± 0.61	1.86 ± 0.59		0.042		
Minimum SA, mm^2^	6.29 ± 2.99	4.81 ± 2.11		0.021		
Minimum stent symmetry index	0.72 ± 0.07	0.58 ± 0.08		0.013		
Mean stent symmetry index	0.83 ± 0.03	0.72 ± 0.03		0.022		

*DoCE* device-oriented composite endo points, *CN* calcium nodule, *CKD* chronic kidney disease, *HD* hemodialysis, *PAD* peripheral artery disease, *RCA* right coronary artery, *OA* orbital atherectomy system, *RA* rotational atherectomy, *PSBA* scoring balloon angioplasty, *MSA* minimum stent area, *MLD* minimum lumen diameter

Univariate analyses were performed to compare patients with and without DoCE, followed by multivariate Cox regression analysis of significant variables. The presence of CN was significantly higher in the DoCE group (25.9% vs. 12.2%, OR: 2.518, p = 0.001), as was insulin use (14.7% vs. 4.3%, OR: 3.778, p < 0.0001), and HD (56.9% vs. 17%, OR: 6.465, p < 0.0001). Additionally, RCA lesions were more common in patients with DoCE (56% vs. 26.1%, OR: 23.611, p < 0.0001), along with higher rates of stent malapposition (63.8% vs. 44.8%, OR: 2.172, p < 0.0001). Univariate analysis revealed significant associations between DoCE and several factors, including CN, male gender, insulin use, HD, PAD, RCA lesions, and stent malapposition.

Multivariate analysis identified independent predictors of DoCE, including HD (OR: 4.318, p < 0.0001), RCA lesions (OR: 3.207, p < 0.0001), insulin use (OR: 1.764, p = 0.037), and CN (OR: 1.806, p = 0.038). The presence of calcium cracks showed a protective effect (OR: 0.657, p = 0.036).

The comparison of clinical findings between patients with and without DoCE in each group is shown in Table 6. In both CC and EC groups, the frequency of calcium cracks and calcium shoulder dissection was significantly lower, and the stent length was significantly longer, in patients with DoCE. Additionally, in both CC and EC groups, acute gain and MSA were significantly smaller in patients with DoCE. In all groups, MSA and minimum SSI were significantly lower in patients with DoCE.

**Table 6:** Comparison between the patients with and without DoCE in each group

	CC N = 273			EC N = 217			CN N = 86		
	DoCE (-) N = 231	DoCE ( +) N = 42	*p*-value	DoCE (-) N = 173	DoCE ( +) N = 44	*p*-value	DoCE (-) N = 56	DoCE ( +) N = 30	*p*-value
Calcium crack, %	80.1	64.3	0.023	13.6	6.9	0.03	5.4	3.3	1
Calcium shoulder dissection, %	49.3	49.5	0.92	91.1	79.2	0.043	57.1	33.3	0.043
Malappostion, %	31.6	42.9	0.159	50.9	65	0.091	82.5	87	0.361
Total stent length, mm	28.9 ± 13.8	31.2 ± 12.9	< 0.0001	30.1 ± 10.9	36.9 ± 10.9	< 0.0001	25.5 ± 12.2	25.4 ± 14.1	0.513
Stent size, mm	2.93 ± 0.39	2.87 ± 0.41	0.832	2.93 ± 0.39	2.88 ± 0.41	0.832	3.33 ± 0.44	3.45 ± 0.42	0.177
Post MLD, mm	2.85 ± 0.37	2.68 ± 0.60	0.002	2.89 ± 0.41	2.59 ± 0.60	0.02	3.27 ± 0.67	3.08 ± 0.59	0.379
Acute gain, mm	2.12 ± 0.61	1.76 ± 0.53	0.03	2.11 ± 0.61	1.76 ± 0.53	0.025	2.44 ± 0.75	2.31 ± 0.68	0.237
Minimum stent area, mm^2^	6.17 ± 2.39	5.21 ± 1.74	0.017	5.45 ± 2.86	4.72 ± 1.68	0.028	7.02 ± 2.43	6.42 ± 1.89	0.027
Minimum stent symmetry index	0.78 ± 0.07	0.61 ± 0.08	0.012	0.63 ± 0.07	0.48 ± 0.09	0.007	0.72 ± 0.09	0.61 ± 0.07	0.034
Mean stent symmetry index	0.85 ± 0.07	0.84 ± 0.04	0.599	0.78 ± 0.03	0.68 ± 0.04	0.036	0.83 ± 0.03	0.82 ± 0.03	0.794

*DoCE* device-oriented composite endo points, *MLD* minimum lumen diameter

## Discussion

In this study, we compared the clinical outcomes of PCI across different morphologies of severely calcified lesions over a median follow-up of 35.4 months. Our results showed that the incidence of DoCE was significantly higher in the CN group compared to the CC and EC groups (CN: 36.0%, CC: 18.3%, EC: 23.5%; p = 0.001), highlighting the unique challenges posed by CN lesions due to their complex morphology and difficulty in achieving effective calcium modification.

Key findings include: (1) The highest incidence of DoCE was observed in the CN group. (2) Calcium cracks were rarely observed in EC and CN groups compared to CC, with significantly smaller SSI in the EC and CN groups. (3) In patients with DoCE, both EC and CN groups showed lower frequencies of calcium cracks and shoulder dissections, and significantly smaller MSA and SSI compared to patients without DoCE. (4) CN, insulin use, HD, RCA lesions, and calcium cracks were identified as independent predictors of DoCE.

Significant differences in MSA and SSI between patients with and without DoCE underscore the critical role of stent expansion in preventing DoCE in severely calcified lesions [16]. However, achieving adequate dilation remains challenging. Calcium crack formation is essential for optimal PCI outcomes as it improves stent expansion and lumen gain [4, 6]. Our findings consistently indicate that calcium cracks are the sole independent predictor of reduced DoCE. Calcium cracks are crucial for improving stent expansion and reducing DoCE. Therefore, enhancing calcium crack formation should be a primary focus during lesion preparation to optimize PCI outcomes.

### For EC lesions

Despite the use of RA, OA, and SBA in approximately 60% of EC lesions, calcium cracks were infrequently observed. Luminal gain in EC lesions with a calcium arc < 270° can be achieved by stretching the noncalcified quadrant; however, this approach does not result in significant calcium modification and may lead to calcium shoulder dissections. Our findings showed a higher frequency of calcium shoulder dissections in EC lesions compared to CC lesions, with poorer stent expansion in those with DoCE. Effective calcium modification remains a challenge in EC lesions, and while shoulder dissections may aid in stent expansion, careful management is required due to the increased risk of coronary artery perforation. Devices like IVL may offer a potential solution by providing more controlled calcium modification [17].

### For CN lesions

Since Xu first reported the clinical outcomes of CN [18], subsequent studies have highlighted the complexities associated with CN lesions, including their tendency for unfavorable PCI outcomes [19–21]. In our study, CN lesions without DoCE showed higher frequencies of calcium shoulder dissections and larger MSA and SSI compared to those with DoCE, indicating the need for significant calcium modification. However, creating calcium cracks in CN lesions is challenging due to their complex morphology. Previous studies, including those by Morofuji et al. and Kaihara et al., have identified several mechanisms by which CN lesions contribute to adverse outcomes, such as stent strut fracture and acute recoil [20, 22]. Advanced techniques and further research are needed to address these challenges and improve PCI outcomes in patients with CN lesions.

## Conclusions

We compared the clinical outcomes of PCI using DES between the morphologies of severely calcified lesions. The incidence of DoCE after DES implantation was highest in the CN group followed by the EC and CC groups. CN, insulin use, HD, RCA lesions, and calcium cracks were independent predictors of DoCE. Our findings suggest that incorporating techniques to enhance calcium crack formation could be pivotal in improving clinical outcomes for patients with severely calcified lesions. Future research should focus on developing advanced imaging techniques that integrate with atherectomy devices, such as real-time imaging for precise lesion modification, to further improve PCI outcomes in calcified lesions.

## Limitation

This study had several important limitations. First, this was a single-center retrospective and observational study with predominantly east-Asian population and not a randomized control study. Second, the sequences of the PCI procedure and selection of DES were completely dependent on the operator’s discretion and the patient’s coronary anatomy. Hence, selection bias may have affected the conclusions. Third, CAG and CTCA at 12 months after PCI might have biased the incidence of TLR. Fourth, due to severely calcified lesions in approximately 60% of lesions, the IVUS catheter initially failed to pass through the target lesion and required ablation with atherectomy devices before IVUS. However, IVUS was immediately performed after atherectomy and before angioplasty in all patients. Regardless, we might have missed important information from morphological changes after atherectomy. Fifth, IVUS has limited resolution; It is often difficult to differentiate calcified nodules from eccentric calcification using IVUS, as calcified protrusions can resemble calcified nodules in IVUS observations. These differences may have distinct impacts on prognosis. It also could miss the detection of calcium cracks and calcium shoulder dissection. Optical coherence tomography may provide a better resolution for detecting these findings.

## Data Availability

VUS- Versus OCT-Guided Coronary Stent Implantation: a Comparison of Intravascular Imaging for Stent Optimization (Current Cardiovascular Imaging Reports (2018) 11: 34 10.1007/s12410-018-9475-z) In Vivo Comparison Between Optical Coherence Tomography and Intravascular Ultrasound for Detecting Small Degrees of In-Stent Neointima After Stent Implantation(J Am Coll Cardiol Intv 2008;1:168–73).

## Abbreviations

CKD: Chronic kidney disease
DoCE: Device-oriented composite end points
DM: Diabetes mellitus
DES: Drug-eluting stent
HD: Hemodialysis
IVUS: Intravascular ultrasound
OA: Orbital atherectomy
PAD: Peripheral artery disease
PCI: Percutaneous coronary intervention
RA: Rotational atherectomy
SBA: Scoring balloon angioplasty
TLR: Target lesion revascularization
